# Acute Lead Exposure Increases Arterial Pressure: Role of the Renin-Angiotensin System

**DOI:** 10.1371/journal.pone.0018730

**Published:** 2011-04-11

**Authors:** Maylla Ronacher Simões, Eduardo Hertel Ribeiro, Marcos Vinícius A. Vescovi, Honério C. de Jesus, Alessandra S. Padilha, Ivanita Stefanon, Dalton V. Vassallo, Mercedes Salaices, Mirian Fioresi

**Affiliations:** 1 Physiological Sciences Post-Graduation Program, Federal University of Espírito Santo, Vitoria, Espírito Santo, Brazil; 2 Department of Chemistry, Federal University of Espírito Santo, Vitoria, Espírito Santo, Brazil; 3 Department of Nursing, Federal University of Espírito Santo, Vitoria, Espírito Santo, Brazil; 4 Health Science Center of Vitória-EMESCAM, Vitória, Espírito Santo, Brazil; 5 Department of Pharmacology, Universidad Autonoma de Madrid, Madrid, Spain; University of Giessen Lung Center, Germany

## Abstract

**Background:**

Chronic lead exposure causes hypertension and cardiovascular disease. Our purpose was to evaluate the effects of acute exposure to lead on arterial pressure and elucidate the early mechanisms involved in the development of lead-induced hypertension.

**Methodology/Principal Findings:**

Wistar rats were treated with lead acetate (i.v. bolus dose of 320 µg/Kg), and systolic arterial pressure, diastolic arterial pressure and heart rate were measured during 120 min. An increase in arterial pressure was found, and potential roles of the renin-angiotensin system, Na^+^,K^+^-ATPase and the autonomic reflexes in this change in the increase of arterial pressure found were evaluated. In anesthetized rats, lead exposure: 1) produced blood lead levels of 37±1.7 µg/dL, which is below the reference blood concentration (60 µg/dL); 2) increased systolic arterial pressure (Ct: 109±3 mmHg vs Pb: 120±4 mmHg); 3) increased ACE activity (27% compared to Ct) and Na^+^,K^+^-ATPase activity (125% compared to Ct); and 4) did not change the protein expression of the α1-subunit of Na^+^,K^+^-ATPase, AT_1_ and AT_2_. Pre-treatment with an AT_1_ receptor blocker (losartan, 10 mg/Kg) or an ACE inhibitor (enalapril, 5 mg/Kg) blocked the lead-induced increase of arterial pressure. However, a ganglionic blockade (hexamethonium, 20 mg/Kg) did not prevent lead's hypertensive effect.

**Conclusion:**

Acute exposure to lead below the reference blood concentration increases systolic arterial pressure by increasing angiotensin II levels due to ACE activation. These findings offer further evidence that acute exposure to lead can trigger early mechanisms of hypertension development and might be an environmental risk factor for cardiovascular disease.

## Introduction

Lead is a common environmental contaminant that affects all the organs and systems of the body and causes numerous acute and chronic illnesses [1], [2]. All humans have lead in their body as a result of exposure to exogenous sources [3]. This exposure occurs during the manufacture of ammunition, batteries, sheet lead, solder, ceramic glazes, caulking, bronze plumbing, military equipment, drinking water and some surgical equipment [3], [4]. Experimental and epidemiological studies suggest a close relationship between lead exposure, hypertension and cardiovascular disease [5], [6], [7], [8], [9].

The effects of lead on human health depend on blood levels and on the duration of the exposure. The Agency for Toxic Substances and Disease Registry (ATSDR) considered the reference blood lead concentration level to be 60 µg/dL, and concentrations that exceed these values require removal from lead exposure [10], [11]. Nevertheless, individuals with baseline blood lead levels of 46.8 µg/dL or 67 µg/dL have shown increases in arterial pressure [12], [13].

*In vivo* and *in vitro* studies with animals have shown that chronic lead exposure causes hypertension and cardiovascular disease by altering the renin-angiotensin system due to elevated ACE activity [14]–[16], inhibition of Na^+^, K^+^- ATPase [17], induction of oxidative stress, reduction of nitric oxide bioavailability [1], [18]–[21] and depletion of antioxidant reserves [22]. Lead might also act as a calcium substitute in Ca^2+^-dependent signaling pathways by interacting with calmodulin, protein kinase C (PKC) and calcium-dependent potassium channels [23], [24] and stimulating vascular smooth muscle cell proliferation [25]. Certain peripheral and central nervous system mechanisms such as increased sympathetic nerve activity, reduced baroreflex sensitivity and reduced parasympathetic tone have also been implicated in chronic lead-induced hypertension [26], [27], [28].

The effect of chronic exposure to lead on arterial pressure was evaluated by several studies. Carmignani et al. [14], [27], Sharifi et al. [15] and Roncal et al. [29] showed that arterial pressure was significantly increased by chronic exposure to lead. However, few reports have evaluated effects of acute exposure to this metal on arterial pressure. Our group found that acute administration of high lead concentration (100 µM) reduces myocardial contractility [30] and affects the endothelium, releasing cyclooxygenase-derived vasoconstrictors and involving reactive oxygen species [31]. However, rats acutely exposed to lead below the reference blood concentration showed an increase in left ventricular systolic pressure [32].

The current study aimed to explore the effects of acute lead exposure on arterial pressure and to elucidate the mechanisms involved in the very early development of lead-induced hypertension in rats. We accordingly developed an experimental model of acute lead exposure in rats that produces blood lead levels below the reference blood concentration [10], [33]. We then analyzed the effects of this treatment on: *1*) the renin-angiotensin system; *2*) Na^+^, K^+^- ATPase activity; and *3*) the participation of the autonomic reflexes in the increased arterial pressure that occurs in response to lead exposure. Our findings provide the first evidence that acute exposure to lead causes an increase in arterial pressure that is due to increased renin-angiotensin system activity.

## Materials and Methods

### Animals

The studies were performed on 64 male Wistar rats (280–330 g). All experiments were conducted in compliance with the guidelines for biomedical research as stated by the Brazilian Societies of Experimental Biology (Protocols numbers 003/2007). The rats were housed at constant room temperature, humidity, and light cycle (12∶12-hr light-dark) with free access to water and were fed rat chow *ad libitum*.

The protocols were performed with anesthetized rats due to the duration of the experiment and the necessity of maintaining stable arterial pressure. To investigate the acute effects of lead on arterial pressure, a bolus dose of lead (320 µg/kg) was injected intravenously. Lead levels were measured in blood by dilution with a polymer (Triton X-100) and samples were measured in triplicate by atomic absorption spectrometry (AAS5 EA, Carl Zeiss, Germany) as previously described [34]. The detection limit of this equipment is 0.5 µg/dL.

### Hemodynamic Measurements

The rats were anesthetized with urethane (1.2 mg/kg IP), and the carotid artery and jugular vein were cannulated with a polyethylene catheter (PE-50/*Clay-Adams*) and filled with heparin (50 U/ml) in saline. The cannulas were connected to pressure transducers (TSD 104A- Biopac) connected to a preamplifier and to an acquisition system (MP 30 Biopac Systems, Inc; CA) for pressure measurements. The following parameters were analyzed: systolic (SAP) and diastolic (DAP) arterial pressure and heart rate (HR).

All animals (n = 10) were followed up for 120 min and SAP, DAP and HR were recorded before (control condition – time 0) and at 30, 60, 90 and 120 min after lead administration. To ensure that the effects were not dependent on time, a time control experiment (n = 5) was performed under the same conditions with the administration of distilled water. After these protocols, the heart and plasma were removed and stored at −80°C until being used for biochemical measurements.

To assess the participation of the renin-angiotensin system in the blood pressure increase induced by lead exposure, an angiotensin converting enzyme (ACE) inhibitor (enalapril maleate, 5 mg/kg) and an AT_1_ receptor antagonist (losartan, 10 mg/Kg) were used. To assess the possible influence of the autonomic reflexes on arterial pressure, we also performed a co-treatment with the ganglionic blocker hexamethonium (20 mg/Kg). The animals were anesthetized with urethane (1.2 mg/Kg IP), their carotid artery was cannulated to measure arterial parameters and their jugular vein was cannulated for drug infusion. After 20 min of arterial pressure stabilization, enalapril maleate (n = 5), losartan (n = 7) or hexamethonium (n = 9) was injected intravenously and after 30 min the following parameters were measured: SAP, DAP and HR. In the same animal, lead was injected intravenously and these data were measured after 1 h of exposure. During the measurement of arterial pressure, the value reached by the systolic blood pressure after 60 min of exposure was not different from that at 120 min. Given this, the influence of other drugs on arterial pressure were investigated only during 60 min of exposure to lead.

### Biochemical Measurements

#### Plasma ACE activity

The effect of lead on plasma angiotensin converting enzyme (ACE) activity was determined as previously described [35], [36], after 120 min of exposure. Briefly, triplicate purified plasma samples (3 µL) were incubated with 40 µL of assay buffer containing 5 mM Hip-His-Leu (Hippuryl–Histidine–Leucine, ACE substrate) (Sigma Chemical) in 0.4 M sodium borate buffer and 0.9 M NaCl pH 8.3 for 15 min at 37°C. The reaction was stopped by adding 190 µL of 0.34 N NaOH. The product, His–Leu, was measured fluorometrically at 365 nm excitation and 495 nm emission with a fluoro-colorimeter (Synergy 2, Biotek, U.S.A.) after the addition of 17 µL of o-phthaldialdehyde (2%) in methanol. To correct for the intrinsic fluorescence of the plasma, blanks were included by adding Hip-His-Leu, NaOH and o-phthaldialdehyde. The activity calculations were based on Michaelis-Menten first-order kinetics. A calibration curve with ACE substrate was included in each plate (n = 19).

### Na+, K+-ATPase activity

To determine if lead exposure for 120 min was capable of affecting Na^+^, K^+^-ATPase activity, the enzymatic material was extracted as previously described [37]. The heart (n = 10) was homogenized in a solution containing 20 mM Tris-HCl and 1 mM EDTA, pH 7.0. The homogenized tissue was centrifuged at 8,800 rpm for 20 min and the precipitate was discarded. To the supernatant, the same volume of the solution was added and it was centrifuged at 10,000 rpm again for 1 hr. The precipitate was resuspended in 20 mM Tris-HCl pH 7.2 to a final volume of 400 µL.

Na^+^,K^+^-ATPase activity was assayed by measuring Pi liberation from 3 mM ATP in the presence of 125 mM NaCl, 3 mM MgCl_2_, 20 mM KCl and 50 mM Tris-HCl (pH 7.5). The enzyme was preincubated for 5 min at 37°C and the reaction was initiated by adding ATP (30 mM). Incubation times and protein concentration were chosen in order to ensure the measurements were made in the linear part of the reaction. The reaction was stopped by the addition of 200 µL of 10% trichloroacetic acid. Controls containing enzyme preparation added after the addition of trichloroacetic acid were used to correct for non-enzymatic hydrolysis of the substrate. All samples were in triplicate. The specific activity was reported as nmol Pi released per min per mg of protein unless otherwise stated. The specific activity of the enzyme was determined in the presence and absence of 5 mM ouabain. Protein concentrations were measured using the Bradford method [38] with bovine serum albumin as the standard. The Na^+^, K^+^-ATPase activity is the difference between the activity with and without ouabain in µmol fluorescein (mg protein)^−^1 h^−^1.

### Western blot analysis

After the experiments, the hearts were homogenized and proteins (80 µg) were separated by 10% SDS-PAGE gels for AT_1_, AT_2_ and the α-1 Na^+^, K^+^-ATPase subunit. The proteins were transferred to nitrocellulose membranes, which were incubated with mouse monoclonal antibodies for AT_1_ (1∶500, Sigma Chemical, CO, St Louis, USA), AT_2_ (1∶500, Sigma Chemical, CO, St Louis, USA) or Na^+^, K^+^- ATPase α-1 (1∶500, Millipore, San Francisco, U.S.A.). After being washed, the membranes were incubated with anti-mouse (1∶5000, Sigma Chemical, Co, St Louis U.S.A.) immunoglobulin antibody conjugated to horseradish peroxidase. After being washed thoroughly, immunocomplexes were detected using an enhanced horseradish peroxidase/luminal chemiluminescence system (ECL Plus, Amersham International, Little Chalfont, UK) and film (Hyperfilm ECL International). Signals on the immunoblot were quantified with the National Institutes of Health Image V1.56 computer program. The same membrane was used to determine α-actin expression using a mouse monoclonal antibody for α-actin (1∶5000, Sigma Chemical, CO, St. Louis, USA), and after being washed, it was incubated with anti-mouse (1∶5000, Sigma Chemical, Co, St Louis U.S.A.). All reagents for western blotting were purchased from Sigma Chemical Co.

### Drugs Used

The following drugs were used: heparin (Roche Q.F.S.A., Brazil), urethane, bovine serum albumin, lead acetate, hexamethonium hydrochloride, losartan, enalapril maleate and ouabain (Sigma Chemical Co., USA). All other reagents used were of analytical grade from Sigma (St Louis, USA) and E. Merck (Germany).

### Data analysis and statistics

All values are expressed as the mean ± SEM of the number of animals used in each experiment. The results were analyzed using the completely randomized Student's t-test and one-way ANOVA. When ANOVA showed a significant treatment effect, Tukey's *post hoc* test was used to compare individual means. Differences were considered statistically significant at p<0.05. The data was analyzed and the figures were plotted with GraphPad Prism™ (Version 2.0, GraphPad Software, USA).

## Results

### Effect of lead exposure on hemodynamic parameters

The effect of acute exposure to lead on hemodynamic parameters was assessed in anesthetized rats, as shown in Table 1. Lead caused a significant increase in SAP after 60 min. However, no significant change in DAP or HR were observed. The blood lead level 2 h after exposure was 37±1.7 µg/dL (n = 12). Control rats (n = 4) had levels below the detection limit.

**Table 1:** Hemodynamic parameters upon acute lead exposure.

	Ct (0 min)	30 min	30 min		30 min		30 min	
**SAP**(mmHg)	108±3	113±3	118±3*	118±3*		120±4*		
**DAP**(mmHg)	60±3	60±3	61±3	62±3		63±4		
**HR**(bpm)	334±13	348±14	370±27	369±23		365±16		

SAP- systolic arterial pressure, DAP- diastolic arterial pressure, HR- heart rate, Ct- Control. The results are expressed as the mean ± SEM.

*p<0.05 compared with controls (time 0); n = 10.

### Role of the renin angiotensin system in the arterial pressure increase induced by lead exposure

**Figure 1:**
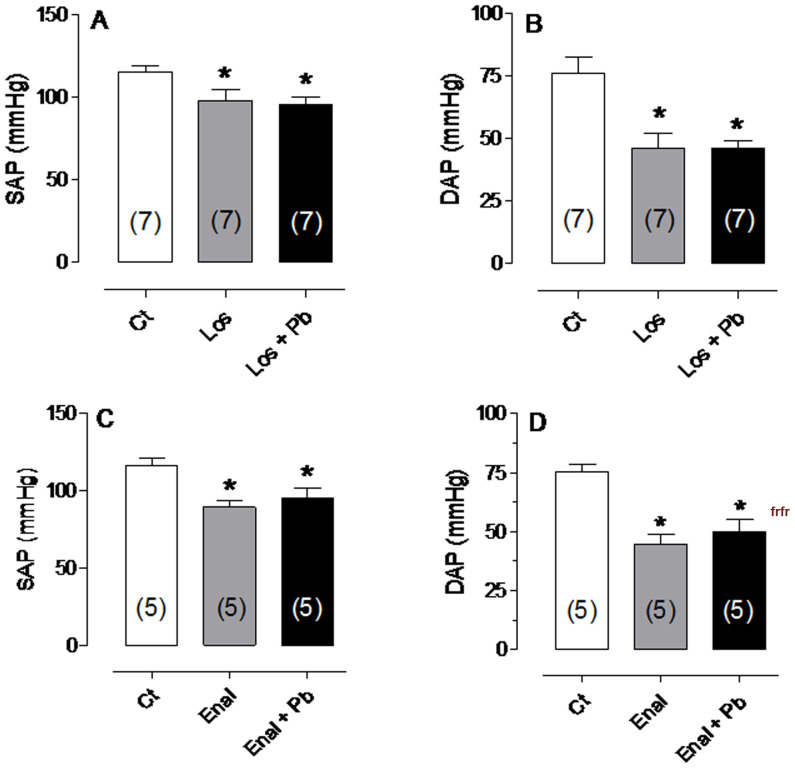
Changes in arterial pressure. Changes in systolic arterial pressure-SAP (A and C) and diastolic arterial pressure-DAP (B and D) before (Ct) and after Losartan (Los) or Enalapril (Enal) administration and following lead exposure (Los+Pb; Enal+Pb). A and B show the Losartan protocol; C and D show the Enalapril protocol. *p<0.05 compared with untreated controls. The number of animals used is indicated in parentheses.

Previous reports have demonstrated that chronic lead administration increases arterial pressure and that this is related to renin-angiotensin system activity [15]–[16]. We asked whether acute lead exposure has similar effects on RAS activity in rats. For this, losartan and enalapril were administered (10 mg/Kg and 5 mg/kg IV, respectively) 30 min prior to lead exposure. Losartan and enalapril reduced SAP and DAP (Figure 1.). In addition, SAP and DAP did not change after lead administration in rats previously treated with losartan or enalapril. Pressure values in the presence of lead remained below the control values, showing that lead did not increase arterial pressure after losartan or enalapril administration.

**Figure 2:**
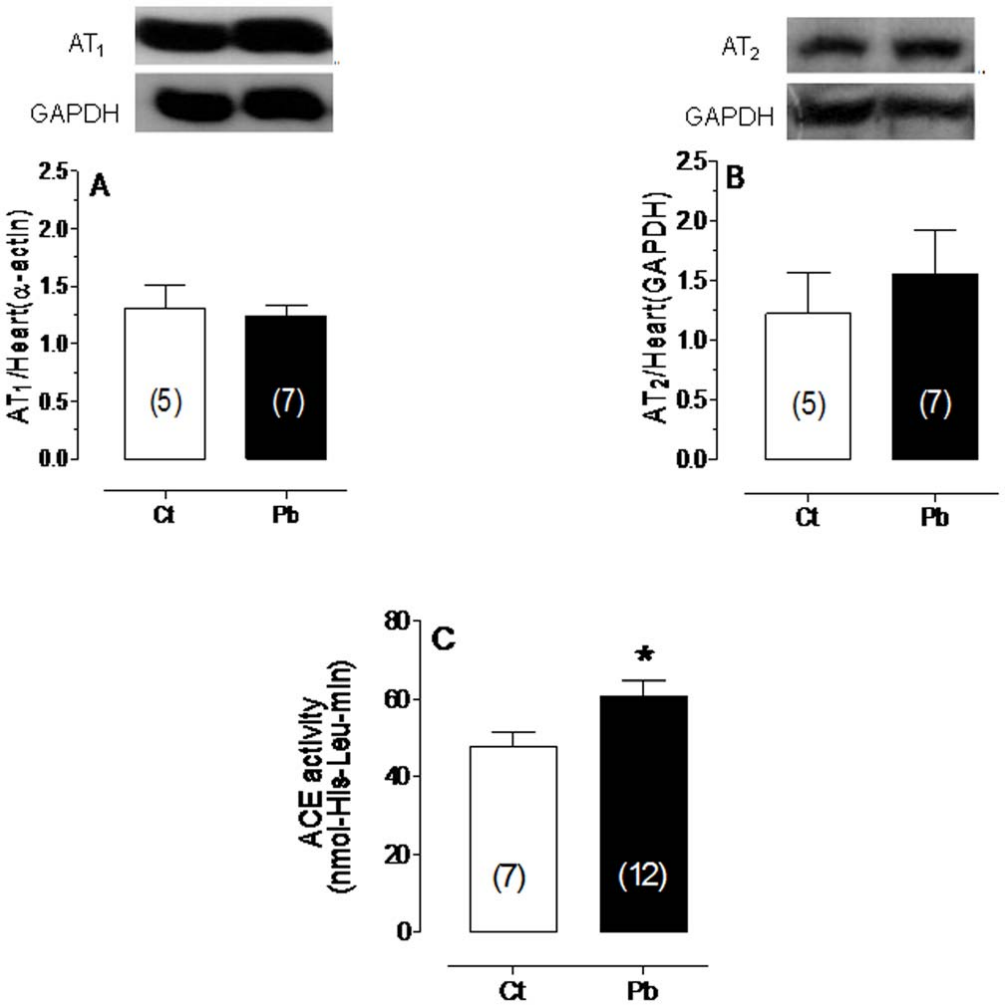
Effects of lead on protein expression and ACE activity. Effects of lead exposure on the protein expression of the (A) AT1 and (B) AT2 receptors and on (C) ACE activity. *p<0.05 compared with untreated controls. The number of animals used is indicated in parentheses.

To investigate the possible mechanisms underlying the role of the RAS in lead-induced hypertension, plasma ACE activity was measured. ACE activity was higher in lead-treated than in untreated rats (27% relative to Ct) (Figure 2. C). However, lead exposure did not change the expression of the AT_1_ or AT_2_ receptors (Figure 2. A–B).

### Role of the autonomic reflexes in the arterial pressure increase induced by lead exposure

**Figure 3:**
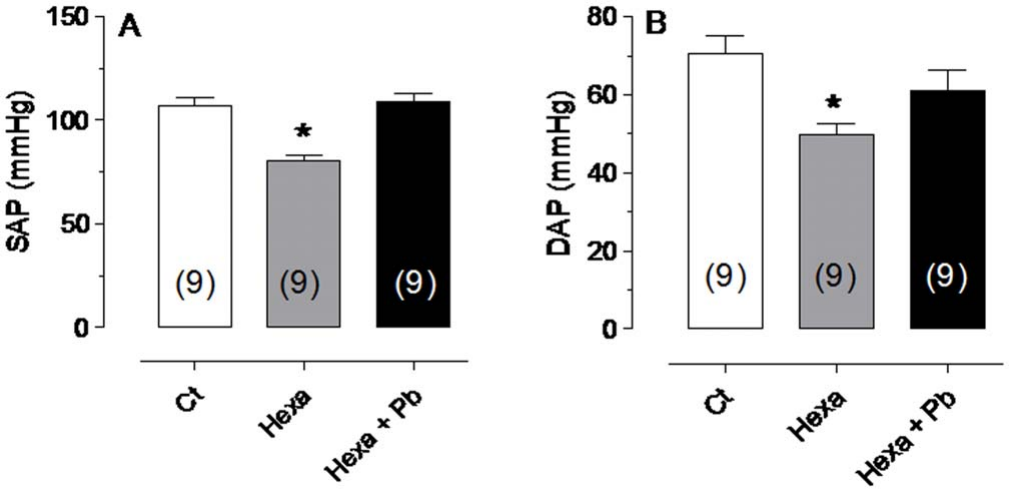
Changes in arterial pressure. Changes in systolic arterial pressure (SAP) and diastolic arterial pressure (DAP) before (Ct) and after Hexamethonium (Hexa) administration and following lead exposure (Hexa+Pb). *p<0.05 compared with untreated controls. The number of animals used is indicated in parentheses.

To determine whether the autonomic reflexes play a role in the pressure changes after lead treatment, the ganglionic blocker hexamethonium was used. Hexamethonium reduced the baseline systolic and diastolic arterial pressure as expected, but these parameters increased after lead treatment, attaining values similar to the control condition, as shown in Figure 3.

### Effect of lead exposure on Na+, K+-ATPase activity

**Figure 4:**
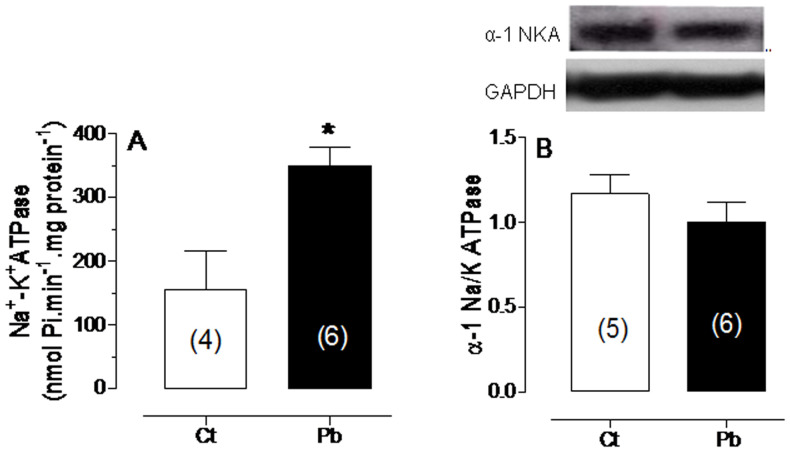
Effects of lead on protein expression and Na^+^, K^+^-ATPase activity. Effects of lead exposure on (A) Na^+^, K^+^- ATPase activity and (B) the protein expression of the α1 subunit of Na^+^, K^+^- ATPase. *p<0.05 compared with untreated controls. The number of animals used is indicated in parentheses.

To evaluate the function of Na^+^, K^+^-ATPase, the activity of this pump was measured and a significant increase was observed in lead-exposed rats (125% relative to Ct) (Figure 4. A). However, the protein levels of the α-1 subunit of Na^+^, K^+^-ATPase were not different between the Ct and Pb groups. These results suggest that acute lead exposure increases the Na^+^, K^+^-ATPase pump's activity, but it does not alter the level of the α-1 subunit.

## Discussion

Lead has been identified as a hazard and a risk factor for hypertension development and other cardiovascular diseases [39]. The Agency for Toxic Substances and Disease Registry (ATSDR) considered the reference blood lead concentration level to be 60 µg/dL [10], [11]. However, the results presented here demonstrate that acute administration of lead results in a concentration (37 µg/dL) below the blood lead reference. This amount of lead is capable of enhancing systolic arterial pressure without altering diastolic pressure or heart rate. Our results also suggest that lead activates ACE and Na^+^, K^+^-ATPase activity.

It is well established that rats chronically treated with high-concentration of lead show increased arterial pressure. Studies by Carmignani et al. [14] have shown that exposure to 60 ppm of lead in drinking water for 10 months increases systolic and diastolic arterial pressure. Other reports have demonstrated an increase of arterial pressure after 2–8 weeks of exposure to lead at 100 ppm [15]. Roncal et al. [29] showed that exposure to 150 ppm of lead in drinking water for 4 weeks increases arterial pressure.

In our protocol we expected that the administration of 320 µg/Kg, should be diluted in a volume correspondent to a 20% of body weight, approximately 20 ml for 100 g of rat, attaining 160 µg/dL in the extracellular fluid. However, blood concentration was 37 µg/dL suggesting that lead accumulates fast in other tissues.

In accordance with those findings, we demonstrate that acute exposure (2 hours) to lead (37 µg/dL), below the reference blood concentration, also increased SAP. DAP did not change, as also observed by Roncal et al. [29]. Our results also showed that 2 h of lead exposure did not alter heart rate (HR). This finding is consistent with the studies of Carmignani et al. [14], [27], who upon investigating the effects of chronic exposure to this metal demonstrated increased cardiac inotropism but no changes in heart rate. Boscolo and Carmignani [26] also showed an increase in arterial pressure and cardiac inotropism with a higher dose of lead, but heart rate was not modified. These authors also demonstrated central sympathetic nervous system hyperactivity, reduced baroreflex sensitivity and vagal hypotonia in rats treated chronically with lead. Similarly, Carmignani et al. [27] reported that lead appears to increase sympathetic nerve activity by central mechanisms. Our results show that the increase of SAP induced by lead was not modified by hexamethonium. This finding also suggests the role of a peripheral effect as an early mechanism in hypertension development.

The effects of lead exposure on the circulating renin-angiotensin system in experimental animals appears to vary depending on the dose and duration of lead exposure [1]. Carmignani et al. [14] found a significant increase in plasma angiotensin-converting enzyme (ACE) activity in rats exposed to lead for 10 months. In a subsequent study of young adult rats exposed to lead for 2–8 weeks, Sharifi et al. [15] found a steady rise in ACE activity in the plasma, aorta, kidney and heart. To our knowledge, there are no studies analyzing the participation of the renin-angiotensin system in a rat model of acute exposure to lead. We found an increase of plasma ACE activity in rats acutely exposed to lead. To further investigate the participation of the renin-angiotensin system in the effect of lead on arterial pressure, we used losartan, an AT_1_ receptor blocker, and enalapril, an ACE activity blocker. These drugs reduced the baseline systolic arterial pressure, suggesting a role of angiotensin II in the acute lead-exposure effects. The expression of the cardiac AT_1_ and AT_2_ receptors was also monitored to determine whether lead's effects could result from changes in these receptors. However, we found that lead was not capable of modifying the expression of these receptors.

Karai et al. [40] reported a strong positive relationship between blood lead and erythrocyte Na^+^, K^+^-ATPase activity in lead-exposed workers. Lee et al. [41] showed that the activation of Na^+^, K^+^-ATPase induced a positive inotropic effect. This was attributable to increased Ca^2+^ influx through L-type Ca^2+^ channels and subsequent sarcoplasmatic reticulum Ca^2+^ release via activation of the Src/Erk1/2 signaling cascade. Our results showed an increase of Na^+^, K^+^-ATPase activity that was independent of the increased expression of the alpha-1 subunit of this pump. Brock et al. [42], Muscella et al. [43], and Zhang and Mayeux [44] demonstrated a sustained increase in Na^+^, K^+^-ATPase activity caused by Angiotensin II. Thus, the increase of ATPase activity we found in our experimental conditions could be associated with the activation of RAS also found in this study.

However, as described years ago, NKA activity in the heart also helps to synchronize contractile activity generating a positive inotropic effect. This synchronization occurs because the cardiac myocytes gain a better resting potential. A slight hyperpolarization occurs resulting in a better fast component generation and consequently a synchronized contraction [45]. Moreover, as previously reported [46], in the vessels lead increases NKA activity, which reduces vascular tone avoiding an increase of DAP. Based on this we believe that the increased NKA activity in the vasculature is the reason to counteract a vasoconstrictor action.

Although observing several similarities among our findings and the findings obtained with chronic treatments it is necessary to consider one fact. Acute events trigger cellular responses usually in a different direction. For example, an increased β-adrenergic stimulation produces, after a time, downregulation of these receptors. In such case a chronic response is inverted compared to the acute one. Indeed, we found in cardiac isolated preparations chronically treated with lead a reduction of the inotropic response to isoproterenol. This was interpreted by the results from Carmignani et al. [27] as a consequence of the increased sympathetic tone. However, we should add that results obtained from acute administration enable us to know which mechanisms might act as a trigger for hypertension. Considering that acute responses are different from those observed with chronic treatments they will enable a better understanding of the natural history of lead-induced hypertension and, consequently, its treatment.

In summary, we found that acute exposure to lead induced an increase in systolic arterial pressure that was associated with increased angiotensin II levels due to ACE activation. These findings also indicate that blood lead concentrations lower than the reference concentration are a risk factor capable of affecting cardiovascular function. Thus, the results presented here provide guidance for revising the lead concentrations considered to be safe and to be toxic.
